# The assessment of the relationship between personality, the presence of the 5HTT and MAO-A polymorphisms, and the severity of climacteric and depressive symptoms in postmenopausal women

**DOI:** 10.1007/s00737-015-0497-0

**Published:** 2015-02-07

**Authors:** Anna Jurczak, Małgorzata Szkup, Sylwia Wieder-Huszla, Anna Grzywacz, Agnieszka Samochowiec, Beata Karakiewicz, Jerzy Samochowiec, Elżbieta Grochans

**Affiliations:** 1 Department of Nursing, Pomeranian Medical University of Szczecin, 48 Żołnierska St., 71-210 Szczecin, Poland; 2 Department of Psychiatry, Pomeranian Medical University in Szczecin, 26 Broniewskiego St., 71-460 Szczecin, Poland; 3 Department of Clinical Psychology, Institute of Psychology, University of Szczecin, 71-79 Krakowska, 71-017 Szczecin, Poland; 4 Department of Public Health, Pomeranian Medical University of Szczecin, 48 Żołnierska St., 71-210 Szczecin, Poland

**Keywords:** Gene polymorphism, Postmenopausal, Personality

## Abstract

The purpose of this study is to determine the relationship between personality, the serotonin transporter (5HTT) and monoamine oxidase A (MAO-A) polymorphisms and the severity of climacteric and depressive symptoms in postmenopausal women. The study involved 272 healthy postmenopausal women from Poland. This survey-based study was performed using the following: the Beck Depression Inventory for depressive symptoms, the Blatt–Kupperman Menopausal Index and the Neuroticism-Extroversion-Openness-Five Factor Inventory for personality. A polymerase chain reaction was employed to identify the DNA polymorphisms. The women were aged 55.4 ± 5.5 years on average. Significant correlations were proved between the allele frequency of the 30-bp variable-number tandem repeat (VNTR) polymorphism in the MAO-A promoter region and the incidence of depressive symptoms in the women analysed (*p* ≤ 0.05), as well as between the severity of climacteric symptoms in the postmenopausal women and the allele frequency of the polymorphism in the 5HTT gene (the 5HTT ‘s’ variant) (*p* ≤ 0.05). There was a significant correlation between the severity of climacteric and depressive symptoms (*p* < 0.001). (1) The severity of climacteric and depressive symptoms depends on personality traits. (2) Personality traits are biologically determined, and the level of their expression is associated with the 5HTT polymorphism. (3) Identification of homogeneous groups of women having predispositions to depressive and severe climacteric symptoms may help to implement early prevention programmes for this group of recipients.

## Introduction

According to McCrae’s theory based on the Big Five factors, called the Five Factor Model, personality traits are biologically determined, stable and unchangeable throughout adult life irrespective of a person’s gender, race and culture. Being of adaptive significance for humans, basic personality traits can be measured using the Neuroticism-Extroversion-Openness-Five Factor Inventory (NEO-FFI) (McCrae and Costa 1985a; 1985b). As said by McCrae, the five fundamental dimensions of personality are the following: neuroticism, extroversion, openness to experience, agreeableness and conscientiousness.

Extroversion reflects individual differences in the level, source and direction of mental energy (Caligiuri 2000). Agreeableness is marked by friendliness, positive attitudes towards people and willingness to co-operate with others. Its components are the following: confidence, straightforwardness, altruism, submissiveness, modesty and a tendency to be moved (Graziano and Tobin 2009). Conscientiousness, on the other hand, concerns people’s ability to organise themselves, their perseverance and motivation to achieve goals and their attitudes towards work. This domain incorporates such features as follows: competence, reliability, a tendency to keep everything in order, striving for achievements, self-discipline and deliberation (Bogg and Roberts 2004). Neuroticism or emotional stability is a dimension which is, to a large extent, genetically determined. Neurotic people display maladjusted behaviours, since they do not always control their impulses. On the other hand, individuals who score low on neuroticism are more emotionally stable, relaxed and less reactive to stress. Components of neuroticism are the following: anxiety, anger, depression, impulsiveness, oversensitivity and excessive self-criticism (McCrae and Costa 1985a; 1985b). Openness to experience is a dimension which describes cognitive curiosity, tolerance for novelty and a tendency to search for experiences and perceive them as valuable. Openness to experience is characterised by the following: imagination, aestheticism, feelings, actions, ideas and values (McCrae and Costa 1985a; 1985b).

A number of studies have been carried out to assess the relationship between depressive symptoms and temperament, understood as a set of inherited, or genetically determined, personality traits. In accordance with Buss and Plomin’s assumptions, so defined, temperament is a basis for personality growth and development (Buss and Plomin 1984). Varied activity of the dopaminergic, serotonergic and noradrenergic systems accounts for individual differences in temperament, while genetic factors are responsible for differences in the functioning of these systems (Ebstein et al. 1997; Lesch et al. 1996; Noble et al. 1998; Samochowiec et al. 2001). Genetic factors may contribute to a risk of some mental disorders. Available studies confirm that depression and anxiety disorders develop on the background of neuroticism and harm avoidance (Hettema et al. 2006; Kendler et al. 2006; Smoller et al. 2005). It is assumed that neurobiological determinants of temperament are less complex than those of mental disorders. Nevertheless, the contribution of genetic variants and early environmental influences significantly contribute to the development of temperament traits (Rybakowski 2007; Samochowiec et al. 2000).

Genes in the serotonergic pathways are regarded as candidate genes due to a well-documented role of serotonin (5-HT) in the etiopathogenesis of mood disorders. One of the most commonly studied genes from this group is SLC 6A4, a gene encoding the serotonin transporter (5HTT). The 5HTT is encoded by a single gene on the 17q12 chromosome. This gene polymorphism is characterised by the insertion or deletion of a 44-bp fragment and is associated with the diversified gene transcriptional activity. A short allele with a 44-bp deletion is associated with a thrice lower transcriptional activity than a long allele with a 44-bp insertion (Hauser and Dmitrzak–Węglarz 2010). Research results suggest that the presence of allele ‘s’ of this gene entails higher neuroticism and the development of anxiety and mood disorders (Stein et al. 2009). The 5HTT expression is of key significance for the development of neuroticism (Cherek and Lane 1999). The presence of allele ‘s’ of the 5HTT gene results in a lower expression of the serotonin transporter and, consequently, a lower flexibility of serotonergic neurotransmission, which in turn leads to higher impulsiveness. There are several studies investigating the influence of the serotonergic manipulation on impulsive behaviours. In most cases, a decrease in serotonergic function was accompanied by higher impulsivity (Lesch and Gutknecht 2005; Murphy et al. 2002; Walderhaug et al. 2002).

The monoamine oxidase A (MAO-A) gene may be also responsible for an inclination to depression. Sabol et al. (1998) were the first to describe the MAO-A polymorphism, which is a variable-number tandem repeat (VNTR) polymorphism in the MAO-A promoter region. It consists of a 30-bp repeated sequence, which can be present in 3, 3.5, 4 and 5 copies (Black et al. 1991). Allele ‘3’ is associated with a lower gene transcriptional activity, while alleles ‘3.5’, ‘4’ and ‘5’ are related to a higher MAO-A activity (Denney et al. 1999). In their previous research performed on a group of 630 women, the authors demonstrated that the serotonin transporter-linked polymorphic region (5HTTLPR) gene polymorphism contributes to climacteric symptoms in postmenopausal women. Furthermore, the results allowed the conclusion that the Blatt–Kupperman Menopausal Index is an instrument which can not only be used for the measurement of the severity of climacteric symptoms but also the early detection of perimenopausal women at the risk of developing depressive symptoms (Grochans et al. 2013).

Menopause is the last menstrual bleeding in a woman’s life, occurring as a consequence of the complete loss of ovarian follicles followed by the cessation of menstrual bleeding for at least 1 year (Taechakraichana et al. 2002; Word Health Organization 1996). In different parts of the world, women reach menopause between the ages of 48 and 52 on average (Kaczmarek 2007). Climacteric symptoms seriously affect the quality of women’s lives, and in some cases, medical intervention to alleviate them is necessary (Chen et al. 2013). Women spend 30 % of their lives in the postmenopausal period; therefore, health problems that they experience after last menstruation have become a serious challenge for modern medicine and social sciences (Broekmans et al. 2009; Burger et al. 2007; Dorland et al. 1998; Drummond et al. 2002; Henderson and Sherwin 2007; Thurston et al. 2008; 2009). The age of menopause has considerable effects on health. Early menopause onset entails a higher risk of cardiovascular diseases, osteoporosis and higher mortality. Late menopause onset, on the other hand, may enhance a risk of breast, ovarian and endometrial cancers (Chen et al. 2013).

Hormonal changes, especially oestrogen deficiency, observed in menopausal women are responsible for numerous clinical symptoms (Grycewicz and Cypryk 2008). The majority of women (approximately 90 %) complain of psychological disorders, manifested, among other things, by sudden mood changes, difficulties in coping with stress of daily living, depressive states, getting tired quickly, nervousness, irritation, poor concentration, deterioration of memory, somatic disorders which do not respond to treatment and full-blown depression (Jagielska et al. 2007). The transition to menopause represents a period of risk for depressive symptoms (Mauas et al. 2014).

The purpose of this study is to determine the relationship between personality, the presence of the 44-bp VNTR polymorphism in the 5HTT (SLC 6A4) promoter region, the 30-bp VNTR polymorphism in the MAO-A promoter region and the severity of climacteric and depressive symptoms in the homogeneous groups of postmenopausal women.

## Materials and methods

### Subjects

The study involved 272 healthy women from northern Poland who had their last menstrual period at least 1 year before the study. These women did not abuse alcohol or cigarettes, or benzodiazepines; had not been diagnosed as having endocrinological, cancerous or mental diseases and had undergone neither hysterectomy nor oophorectomy. The criteria for inclusion in the study were the following: a normal cervical smear result, a normal mammography result, no history of thyroid or cancerous diseases and no psychiatric treatment until that moment. The women who met the above criteria were informed about the possibility of taking part in the study by their gynaecologists. Next, they voluntarily reported to the research centre, where they completed questionnaires and had blood samples taken for analysis. This method guaranteed a 100 % return of questionnaires. After the examination, all patients received their examination results free of charge.

All participants were informed in details about the purpose and the course of the study. Next, they gave their written consent for taking part in the study, for collecting their blood, as well as storing and subjecting it to a genetic analysis. Furthermore, all participants were assured that they could resign from their participation in the further part of the study without the necessity of giving a reason. The written consents of the participants are stored by the corresponding author. The methodology of this study was approved by the Bioethical Commission of the Pomeranian Medical University of Szczecin (permission number KB-0080/187/09).

### Assessments

The first stage of this study was based on a diagnostic survey performed using standard research instruments, namely the Beck Depression Inventory (BDI) for the assessment of depressive symptoms (Beck et al. 1961), the Blatt–Kupperman Menopausal Index (BKMI) measuring the severity of climacteric symptoms (Kupperman et al. 1953) and the Neuroticism-Extroversion-Openness-Five Factor Inventory (NEO-FFI) for the assessment of personality. The latter consists of five scales measuring neuroticism, extroversion, openness to experience, agreeableness and conscientiousness (Costa and McCrae 1976).

Axis I mental disorders of the ICD-10 classification were excluded in all analysed women by means of the PRIME-MD questionnaire and the psychiatric examination (Spitzer et al. 1999).

The second stage of the study was based on genetic tests; the DNA was isolated from the full blood by a salting-out method according to Miller (1988). The polymerase chain reaction (PCR) was used to identify the DNA polymorphisms. The aim of the analysis was to obtain the amplification of the fragment consisting of 2–5 repetitions of the 30-bp VNTR polymorphism in the MAO-A promoter region. The sequences of starters are the following: MAO-A F: 5′ CCC AGG CTG CTC CAG AAA 3′ and MAO-A R: 5′ GGA CCT GGG TTG TGC 3′. The sizes of amplified fragments are the following: 239, 209, 226 and 269 bp. In the analysis of the 5HTT polymorphism, the amplification of the fragment including the 44-bp ins/del in the regulatory sequence (the presence or the lack of 44-bp) was obtained. The sequences of starters are the following: HTT F: 5′ GGC GTT GCC GCT CTG AAT GC 3′ and HTT R: GAG GGA CTG AGC TGG ACA ACC AC 3′. The sizes of amplified fragments are the following: 484 and 528 bp.

### Statistical analyses

A statistical analysis was performed using STATISTICA 7.1 PL. The chi-square independence test was applied to verify the null hypothesis referring to the independence of the analysed variables. Spearman’s rank R correlation coefficient was used to identify and test the strength of a relationship between the ordinal variables. The accepted significance level was *α* ≤ 0.05. The Power calculated in all genetic tests exceeded 0.95 (Power > 0.95).

## Results

The mean age of the women was 55.4 ± 3.5 years. The group was varied in terms of socio-demographic backgrounds: more than a half (53.7 %) of the women had completed secondary education, 36 % higher education, 9.2 % vocational education and 1.1 % primary education. The majority of the women lived in urban areas with a population of more than 100,000 residents (60.3); 9.2 and 7.3 % of the participants lived in rural areas and towns of up to 10,000 residents, respectively; the remainder (23.2 %) lived in towns with no more than 100,000 residents. The vast majority of the participants in the study had life partners (73.2 %). More than a half of the respondents (75 %) were employed (Table 1). According to the BDI, as many as 69.5 % of the women did not show any depressive symptoms; however, these symptoms were diagnosed in 11.8 % of the women. The BKMI diagnosed severe climacteric symptoms in only 11 % of the women analysed, moderate symptoms in 9.9 % of the women and minor symptoms in 15.1 % of the women, and 64 % of the women had no symptoms at all (Table 2).

**Table 1:** The female group structure with regards to the selected socio-demographic data

Socio-demographic data	Number^a^	Per cent^b^	*χ* ^2^ *p* value
Education			
Primary	3	1.1	*χ* ^2^ = 132.495 *p* = 0.000
Vocational	25	9.2	
Secondary	146	53.7	
Higher	98	36.0	
Total	272	100.0	
Place of living			
Country	25	9.2	*χ* ^2^ = 380.571 *p* = 0.000
Town with a population lower than 10 thousand	20	7.3	
Town with a population between 10–100 thousand	63	23.2	
Town with a population higher than 100 thousand	164	60.3	
Total	272	100.0	
Marital status			
In relationship	199	73.5	*χ* ^2^ = 170.462 *p* = 0.000
Single	73	26.5	
Total	272	100.0	
Professional status			
Active	204	75.0	*χ* ^2^ = 383.722 *p* = 0.000
Inactive	68	25.0	
Total	272	100.0	

^a^The number of respondents in the subgroup

^b^The percentage share

**Table 2:** The female group structure with regard to depression and climacteric symptoms

Medical data	Number	Per cent
The Beck Depression Inventory		
No depression	189	69.5
Mild depression	51	18.7
Moderate depression	22	8.1
Severe depression	10	3.7
Total	272	100.0
Climacteric symptoms—according to Blatt–Kupperman		
No climacteric symptoms	174	64.0
Mild symptoms	41	15.0
Moderate symptoms	27	10.0
Severe symptoms	30	11.0
Total	272	100.0

Personality structure according to the NEO-FFI demonstrated that most women obtained average scores for all personality dimensions (40–82 % of women in particular domains), which may prove that the sample was well matched (Table 3).

**Table 3:** Personality structure according to the NEO-FFI

NEO-FFI	Low		Average		High		Total	
	*n*	%	*n*	%	*n*	%	*N*	%
Neuroticism	92	34	111	41	69	25	272	100
Extroversion	40	15	135	50	97	35	272	100
Openness to experience	35	13	108	40	129	47	272	100
Agreeableness	32	12	135	50	105	38	272	100
Conscientiousness	43	16	223	82	6	2	272	100

*n* number of women in subgroup

Women with high conscientiousness scores according to the NEO-FFI were excluded from the further research due to their insufficient number.

The analysis of the research material revealed a statistically significant relationship between the level of agreeableness according to the NEO-FFI and the genotype distribution of the 5HTT polymorphism (*p* < 0.01). Women with the l/s genotype had low agreeableness scores considerably rarer (15.6 %) and medium scores more frequently (50.0 %) than others. High agreeableness scores were significantly more common among women with the l/l genotype (55.8 %) than with the s/s genotype (9.6 %). There was also a statistically significant relationship between the openness to experience scores and the frequency of alleles of the 44-bp polymorphism in the 5HTT (SLC 6A4) promoter region (*p* ≤ 0.05). Allele ‘l’ was significantly more common in women who scored high on openness to experience than allele ‘s’ (68.2 and 31.8 % respectively). A similar situation was observed in women who obtained average scores (71.2 and 28.8 %, respectively) (Table 4).

**Table 4:** The genotype distribution and the allele frequency of the 44-bp polymorphism in the 5HTT (SLC 6A4) promoter region vs. personality trait levels according to the NEO-FFI

NEO-FFI (level)	Number	Genotype			Allele	
		l/s *n* (%)	s/s *n* (%)	l/l *n* (%)	l *n* (%)	s *n* (%)
Conscientiousness		*χ* ^2^ = 2.9, *p* > 0.05			*χ* ^2^ = 1.59, *p* > 0.05	
Low	43	22 (51.2)	5 (11.6)	16 (37.2)	54 (62.8)	32 (37.2)
Average	221	84 (38.0)	25 (11.3)	112 (50.7)	308 (69.7)	134 (30.3)
High	6	2	3	1	4	8
Agreeableness		*χ* ^2^ = 17.37, ***p*** **< 0.001**			*χ* ^2^ = 5.59, *p* > 0.05	
Low	32	5 (15.6)	7 (21.9)	20 (62.5)	45 (70.3)	19 (29.7)
Average	134	67 (50.0)	16 (11.9)	51 (38.1)	169 (63.1)	99 (36.9)
High	104	36 (34.6)	10 (9.6)	58 (55.8)	152 (73.1)	56 (26.9)
Extraversion		*χ* ^2^ = 3.65, *p* > 0.05			*χ* ^2^ = 1.37, *p* > 0.05	
High	96	36 (37.5)	10 (10.4)	50 (52.1)	136 (70.8)	56 (29.2)
Average	134	52 (38.8)	20 (14.9)	62 (46.3)	176 (65.7)	92 (34.3)
Low	40	20 (50.0)	3 (7.5)	17 (42.5)	54 (67.5)	26 (32.5)
Neuroticism		*χ* ^2^ = 3.04, *p* > 0.05			*χ* ^2^ = 0.29, *p* > 0.05	
High	69	25 (36.2)	11 (15.9)	33 (47.9)	91 (65.9)	47 (34.1)
Average	109	41 (37.6)	14 (12.8)	54 (49.6)	149 (68.3)	69 (31.7)
Low	92	42 (45.7)	8 (8.7)	42 (45.6)	126 (68.5)	58 (31.5)
Openness to experience		*χ* ^2^ = 5.5, *p* > 0.05			*χ* ^2^ = 5.84, ***p*** **≤ 0.05**	
High	129	52 (40.3)	15 (11.6)	62 (48.1)	176 (68.2)	82 (31.8)
Average	106	39 (36.8)	11 (10.4)	56 (52.8)	151 (71.2)	61 (28.8)
Low	35	17 (48.6)	7 (20.0)	11 (31.4)	39 (55.7)	31 (44.3)

Bold values mean a statistical significance

*n* number of women in genotype subgroup, *χ* ^*2*^ Pearson’s chi-square test; *p* level of significance for *χ* ^2^

There was no significant relationship between the scores on the five personality domains according to the NEO-FFI and the distribution of genotypes and the frequency of alleles of the 30-bp VNTR polymorphism in the MAO-A promoter region (Table 5.).

**Table 5:** The genotype distribution and the allele frequency of the 30-bp VNTR polymorphism in the MAO-A promoter region vs. personality trait levels according to the NEO-FFI

NEO-FFI (level)	Number	Genotype			Allele	
		3/3 *n* (%)	3/4 *n* (%)	4/4 *n* (%)	3 *n* (%)	4 *n* (%)
Conscientiousness		*χ* ^2^ = 0.58, *p* > 0.05			*χ* ^2^ = 0.52, *p* > 0.05	
Low	43	5 (11.6)	17 (39.6)	21 (48.8)	27 (31.4)	59 (68.6)
Average	223	30 (13.5)	98 (43.9)	95 (42.6)	158 (35.4)	288 (64.6)
High	6	1	2	3	4	8
Agreeableness		*χ* ^2^ = 4.07, *p* > 0.05			*χ* ^2^ = 4.28, *p* > 0.05	
Low	32	3 (9.3)	13 (40.7)	16 (50.0)	19 (29.7)	45 (70.3)
Average	135	15 (11.1)	56 (41.4)	64 (47.4)	86 (31.9)	184 (68.1)
High	105	18 (17.1)	48 (45.7)	39 (37.2)	84 (40.0)	126 (60.0)
Extroversion		*χ* ^2^ = 2.52, *p* > 0.05			*χ* ^2^ = 1.32, *p* > 0.05	
High	97	13 (13.4)	38 (39.2)	46 (47.4)	64 (33.0)	130 (67.0)
Average	135	18 (13.3)	64 (47.4)	53 (39.3)	100 (37.0)	170 (63.0)
Low	40	5 (12.5)	15 (37.5)	20 (50.0)	25 (31.3)	55 (68.7)
Neuroticism		*χ* ^2^ = 5.2, *p* > 0.05			*χ* ^2^ = 3.05, *p* > 0.05	
High	69	5 (7.2)	30 (43.5)	34 (49.3)	40 (29.0)	98 (71.0)
Average	111	20 (18.0)	44 (39.6)	47 (42.4)	84 (37.8)	138 (62.2)
Low	92	11 (12.0)	43 (46.7)	38 (41.3)	65 (35.3)	119 (64.7)
Openness to experience		*χ* ^2^ = 6.04, *p* > 0.05			*χ* ^2^ = 0.94, *p* > 0.05	
High	129	17 (13.2)	61 (47.3)	51 (39.5)	95 (36.8)	163 (63.2)
Average	108	12 (11.1)	47 (43.5)	49 (45.4)	71 (32.9)	145 (67.1)
Low	35	7 (20.0)	9 (25.7)	19 (54.3)	23 (32.9)	47 (67.1)

*n* number of women in genotype subgroup, *χ* ^*2*^ Pearson’s chi-square test; *p* level of significance for *χ* ^2^

A significant correlation was observed between the severity of depressive symptoms according to the BDI and agreeableness, extroversion and neuroticism according to the NEO-FFI (*p* < 0.001). In two first cases, there was a clear but insignificant correlation between variables. The severity of depressive symptoms correlated negatively with agreeableness and extroversion (the more severe depressive symptoms, the lower agreeableness and extroversion) and correlated highly positively with neuroticism (the more severe the depressive symptoms, the higher the neuroticism) (Table 6). Similar results were obtained in the correlation analysis of the NEO-FFI scores and the severity of climacteric symptoms according to the BKMI (Table 7).

**Table 6:** Correlation between the severity of depressive symptoms according to the BDI and personality traits according to the NEO-FFI

	Number^a^	*r*	*t* (N-2)	*p* value^b^
Depressive symptoms and conscientiousness	272	−0.037	−0.61	>0.05
Depressive symptoms and agreeableness	272	−0.250	−4.24	**<0.001**
Depressive symptoms and extroversion	272	−0.331	−5.80	**<0.001**
Depressive symptoms and neuroticism	272	0.601	12.37	**<0.001**
Depressive symptoms and openness to experience	272	−0.063	−1.03	>0.05

Bold values mean a statistical significance

*r* Spearman’s rank correlation coefficient, *t* (*N*-*2*) test statistics for significance of correlation coefficient *r*

^a^Number of women analysed

^b^Level of significance for *t*-statistics

**Table 7:** Correlation between the severity of climacteric symptoms according to the BKMI and personality traits according to the NEO-FFI

	Number^a^	*r*	*t* (N-2)	*p* value^b^
Severity of climacteric symptoms and conscientiousness	272	−0.091	−1.50	>0.05
Severity of climacteric symptoms and agreeableness	272	−0.166	−2.76	**<0.01**
Severity of climacteric symptoms and extroversion	272	−0.272	−4.64	**<0.001**
Severity of climacteric symptoms and neuroticism	272	0.413	7.45	**<0.001**
Severity of climacteric symptoms and openness to experience	272	−0.076	−1.25	>0.05

Bold values mean a statistical significance

*r* Spearman’s rank correlation coefficient, *t* (*N*-*2*) test statistics for significance of correlation coefficient *r*

^a^Number of women analysed

^b^Level of significance for *t*-statistics

## Discussion

Research findings show that the presence of a high activity 30-bp VNTR polymorphism in the MAO-A promoter region may contribute to the development of such personality traits as impulsivity (Manuck et al. 2000) and neuroticism (Eley et al. 2003), but this was only observed in men. The MAO-A genotypic variation has been associated with variation in aggression, especially in interaction with childhood trauma or other early adverse events (Verhoeven et al. 2012). This study, however, did not demonstrate significant differences in the frequency of genotypes and alleles of the 30-bp VNTR polymorphism in the MAO-A promoter region in relation to particular personality domains. The fact that the relationship between the variables analysed was not confirmed may result from the absence of environmental factors contributing to the expression of personality traits in the study group. Further analyses should take into consideration the influence of gender on the development of particular personality traits in relation to the activity 30-bp VNTR polymorphism in the MAO-A promoter region.

Some reports suggest that the 5HTT polymorphism may influence the level of impulsivity in particular groups of people, ex. allele ‘s’ was more common among those who had attempted suicide (Baca-Garcia et al. 2005) and type 2 alcoholics according to Cloninger’s typology (Hallikainen et al. 1999).

The aim of the study carried out in Estonia was to determine the relationship between the development of impulsivity in youths and the presence of the 5HTTLPR polymorphism combined with the unfavourable impact of the home environment. It was proved that girls receiving less warmth from parents and carrying the 5HTTLPR short allele more often behaved impulsively and without inhibitions. And quite the opposite, family relationships had no effects on the level of impulsivity in girls carrying the long allele. The outcomes of the study imply that the presence of allele ‘s’ may lead to impulsivity due to greater sensitivity to adversities. This problem is much more common among girls than boys (Paaver et al. 2008).

Caspi’s research provided evidence that people with the 5HTTLPR short allele manifested more severe depressive symptoms and more often attempted suicide as a consequence of stressful life events than their homozygous counterparts with the long allele (2003).

The study based on a meta-analysis revealed a small but significant relationship between the 5HTTLPR polymorphism and neuroticism (Schinka et al. 2004; Sen et al. 2004), which is believed to be a risk factor of affective disorders. Thus, it seems very probable that the 5HTTLPR polymorphism is directly associated with a risk of anxiety and mood disorders, which is not necessarily related to its influence on a level of resistance to stress (Stein et al. 2009).

The purpose of the study conducted by Stein et al. was to assess the association between the presence of the 5HTTLPR polymorphism, depressive symptoms measured with the BDI and the level of neuroticism assessed with the NEO-Five Factor Inventory Neuroticism subscale (NEO-N). The analysis did not demonstrate a statistically significant relationship between these variables. It was observed, however, that the 5HTTLPR polymorphism had an influence on psychological resilience. Allele ‘s’ entailed lower resistance to stressful situations (Stein et al. 2009).

On the contrary, the research described in this article proved significant positive correlations between neuroticism and the severity of depressive and climacteric symptoms in postmenopausal women (the higher the neuroticism scores, the more severe the depressive and climacteric symptoms). Previous studies confirmed a higher frequency of the genotype s/s and the allele ‘s’ of the 44-bp VNTR polymorphism in the 5HTT (SLC 6A4) promoter region in women with more severe depressive symptoms and a lower frequency of allele ‘s’ in women without climacteric symptoms (Grochans et al. 2013).

The relationship between depression, extroversion and neuroticism has been analysed in many studies conducted in families of people suffering from depression (Duggan et al. 1995), twins (Kendler et al. 1993), non-clinical (Farmer et al. 2002) and clinical groups (Bienvenu et al. 2004; Cuijpers et al. 2005) and in general population (Cox et al. 2004). The study carried out in Finland showed that neuroticism was strongly related to the occurrence of anxiety and depressive symptoms, while introversion had moderate effects on the development of depression. These findings were clinically confirmed by the respondents’ opinions about the relationship between these personality dimensions and the frequency of mental health problems as well as the necessity of medical care for psychiatric reasons (Jylhä and Isometsä 2006).

The issue of the relationship of genetic effects on personality variables to clinical conditions is well established for personality traits such as neuroticism (being shy, moody, anxious and sad) representing a vulnerability factor for depression (Felten et al. 2011). The study of Paaver et al. indicates that an influence of the MAO activity in thrombocytes on the development of impulsivity depends on the 5HTTLPR genotype. There is also a similar but reverse relationship. Carriers of the 5HTTLPR allele ‘s’ with the low MAO activity had the highest level of impulsivity comparing to the other groups. People with the low MAO activity and the 5HTTLPR l/l genotype had lower levels of impulsivity (Paaver et al. 2007).

Similar results were obtained in the research performed among 197 Taiwanese perimenopausal women. In this study, interaction between neuroticism and extroversion was statistically significant. In the group with higher neuroticism, extroversion correlated negatively with depressive symptoms. In the group with low neuroticism, there was no relationship between extroversion and depressive symptoms. All highly neurotic women were more prone to depression. The climacteric period had some effects on the incidence of depression but they appeared to be insignificant. The results of the study confirm that personality may contribute to the occurrence of depressive symptoms in perimenopausal women (Paaver et al. 2008).

### Limitations

The authors of this study analysed the relationship between personality, the 5HTT and MAO-A polymorphisms and the severity of climacteric and depressive symptoms in 272 postmenopausal women. It would increase the value of this research if the study group was more numerous and environmental factors, potentially contributing to the incidence and severity of depressive symptoms, were taken into consideration in some respondents, especially those genetically predisposed to such disorders. The use of instruments providing information about adverse life events could give a new look at biological and environmental determinants of depressive symptoms occurring in postmenopausal women.

Furthermore, it should be remembered that the findings presented in this article only concern the women without a clinical diagnosis of depression. Perhaps, the inclusion of such women in the study would reveal significantly stronger relationships between the variables analysed than it was observed in this study.

Despite its all limitations, not allowing us to draw the conclusions for the general population, this is an innovative study presenting a new research area and suggesting the direction of further investigation.

## Conclusions

1. The severity of climacteric and depressive symptoms correlated with personality traits, such as agreeableness, extroversion and neuroticism.
2. Some personality traits may be biologically determined, and the level of their expression is associated with the 5HTT polymorphism.
3. Identification of homogeneous groups of women having predispositions to depressive and severe climacteric symptoms may help to implement early prevention programmes for this group of recipients.
